# Effect of Amaranth and Quinoa Flours on Exopolysaccharide Production and Protein Profile of Liquid Sourdough Fermented by *Weissella cibaria* and *Lactobacillus plantarum*

**DOI:** 10.3389/fmicb.2020.00967

**Published:** 2020-05-21

**Authors:** Francesca Valerio, Anna Rita Bavaro, Mariaelena Di Biase, Stella Lisa Lonigro, Antonio Francesco Logrieco, Paola Lavermicocca

**Affiliations:** Institute of Sciences of Food Production, National Research Council of Italy, Bari, Italy

**Keywords:** pseudocereal, fermentation, lactic acid bacteria, protein degradation, chip electrophoresis, bread improvers, viscosity

## Abstract

Exopolysaccharides (EPSs) are known for their positive contribute to the technological properties of many foods, including bakery products. These molecules can be obtained performing piloted fermentation with lactic acid bacteria (LAB). In order to select strains able to produce EPS, a screening test in agar medium containing sucrose, fructose or glucose as carbohydrate source was performed on 21 LAB strains. Results allowed to select 8 *Weissella cibaria*, 2 *Weissella confusa*, and 2 *Leuconostoc* spp. strains as EPS producers only in the presence of sucrose. A further screening in liquid medium enriched with sucrose (10%) (mMRS_S) indicated the *W. cibaria* strain C43-11 as the higher EPS producer. The selected strain was used to develop liquid sourdoughs (LSs) with dough yield (DY) 500, fermented for 15 h and based on wheat flour and wheat gluten or pseudocereals (quinoa or amaranth) in ratio 1:1, in the presence or not of sucrose at 3% (w/w, LS weight), in comparison to *Lactobacillus plantarum* ITM21B, a strain not producing EPS in mMRS_S. Results indicated that the use of pseudocereals favored the EPS production. Formulations were optimized by modifying DY (500 or 250), sucrose concentration (3 or 6%) and flour ratio. LSs were characterized for the content of organic acids (lactic, acetic, phenyllactic, OH-phenyllactic), pH, TTA, EPS, viscosity, total protein degradation and protein pattern. The highest EPS production (20.79 g/kg) and viscosity (1168 mPa s) were obtained in LS (DY 250, sucrose 6%) based on quinoa flour and started with C43-11 strain. The LS was characterized by the presence of phenyllactic and OH-phenyllactic acids, protein degradation by 51.7% and proteins in the range 14–80 kDa. In these conditions, also strain ITM21B was able to produce EPS at level of 4.61 g/kg and to degrade proteins by 53.8% in LS based on wheat and quinoa flours (1:1) (DY250 and sucrose 3%). Therefore, results demonstrated that the use of selected conditions (flour type, DY, sucrose) can stimulate specific attributes of strains making them suitable for production of short fermented (15 h) LSs which can be used as bread improvers.

## Introduction

According to the World Health Organization (WHO) industries should contribute to improve human nutrition by providing high quality foods in terms of nutritional and functional attributes. The requirements for a nutritionally balanced diet have driven food producers to modify processing by exploring high nutritious raw materials and adopting sustainable methodologies that can maximize their health outcome (Norum, 2005; Galli et al., 2017). Information and methodologies are made available by the scientific community involved in research with high technology transfer level often supported by public actions, involving producers that focus at optimizing the organoleptic quality of foods while valorizing their nutrient content. Particularly, the bakery sector is currently exploring biotechnological processes, also combining alternative flours as sources of carbohydrates, proteins, vitamins and minerals to wheat flour, to improve the nutritional, functional and technological quality of products. The interest toward bakery products with reduced fat content and/or processed using protein sources such as non-wheat cereal flours other than ancient grains and legumes, is increased (Nishida et al., 2004; Gobbetti et al., 2019). Traditional cereal-based products can be produced replacing, at least part, wheat flour with pseudocereals, for instance amaranth and quinoa, because of their nutritional and textural features (Coda et al., 2014). Amaranth (*Amaranthus cruentus* or *hypochondriacus*) has a higher mineral and protein content than most cereal grains and a very balanced amino acid composition. Although amaranth grain is a high glycemic ingredient, it is a rich source of unsaturated fatty acids and polyphenols (flavonoids) with cholesterol-lowering and antioxidant activities (Singh and Singh, 2011). The cooking of amaranth improves its digestibility and the absorption of nutrients. Amaranth proteins are composed mainly of three major fractions (albumins, globulins, and glutelins) with little or no storage prolamin. Amarantin is the most important component of globulins constituting its 90% and approximately the 19% of the total grain proteins (Singh and Singh, 2011). Quinoa (*Chenopodium quinoa* Willd.) is a nutritionally well-balanced pseudocereal due to the high protein content that ranges from 12.9 to 16.5% (Bastidas et al., 2016) and essential amino acids (lysine, threonine, and methionine), as well as unsaturated fatty acids (linolenic and linoleic acids), vitamins and minerals (Wang and Zhu, 2016). These pseudocereals are widely used to produce gluten-free products, even if the lack of gluten, that contributes to produce a viscoelastic dough, limits their use in bread-making (Lynch et al., 2018).

To overcome this technological drawback several additives can be used (Houben et al., 2012; Lynch et al., 2018) including hydrocolloids, which are water-soluble, long-chain polysaccharides that bind water and modify the dough viscosity then improving the loaf volume and crumb structure of the breads (Lazaridou et al., 2007; Sciarini et al., 2010; Mir et al., 2016). Hydrocolloids can be added to the dough also as fat replacer in baked goods (Colla et al., 2018). These substances are generally added at 0.1–1% of flour basis to improve the rheological properties of dough (Rosell et al., 2001; Guarda et al., 2004). Hydrocolloids mainly originate from plants or plant seeds (pectin, locust bean gum, and guar gum), but there are similar molecules produced by bacteria, the exopolysaccharides (EPS) with the same properties (Zannini et al., 2016; Lynch et al., 2018).

Depending on their composition and biosynthesis mechanism, EPS can be divided into homopolysaccharides (HoPS) and heteropolysaccharides (HePS) (Galle and Arendt, 2014). Lactic acid bacteria (LAB) can produce both types. HoPS, generally produced by *Weissella, Leuconostoc, Streptococcus, Lactobacillus*, consist of one monosaccharide, either glucose or fructose, with the resulting EPS termed glucans or fructans, respectively (Lynch et al., 2018). Their synthesis includes intracellular and extracellular steps through the activity of glycansucrases (glycosyltransferases) (Badel et al., 2011). Among EPSs, dextran, a homopolysaccharide in which the main unit is composed by α-1-6 glycosidic linkages, can be used in foods since it has been generally recognized as safe (GRAS) by the Food and Drug Administration. As an example, the use of a *Leuconostoc mesenteroides* preparation containing dextran has been authorized by the European Commission as food ingredient in bakery products (Byme, 2001) to improve softness, crumb texture and loaf volume. The polymer dextran is generally produced by *Leuconostoc* and *Weissella* even if it has been synthesized by some *Lactobacillus plantarum* strains (Di Cagno et al., 2006; Das and Goyal, 2014).

HePS are generally produced in lower amounts by mesophilic and thermophilic LAB (*Lactobacillus lactis, Lactobacillus casei, Lactobacillus plantarum, Lactobacillus rhamnosus, Lactobacillus helveticus*, etc.) and are known for their positive role in the rheology, texture and mouthfeel of fermented dairy products (Tallon et al., 2003; Zannini et al., 2016; Şentürk et al., 2020).

A biotechnological approach combining pseudocereals and EPS producing LABs, can improve the overall quality (shelf-life, flavor, dough structure) of final products and their nutritional characteristics (Rühmkorf et al., 2012; Wolter et al., 2014). Authors demonstrated the suitability of pseudocereals as substrate for EPS production by LAB strains. Moreover, the protein hydrolysis operated by LAB determines the formation of several molecules, including amino acids, organic acids, bacteriocins, peptides, etc. (De Vuyst et al., 2009). Furthermore, pseudocereal proteins are generally more accessible to cereal and microbial proteases than wheat since their hydrophilic nature (Dallagnol et al., 2013; Wolter et al., 2014). Therefore, these studies suggest the suitability of pseudocereals to produce sourdough with high nutritional value. Moreover, due to their water holding capacity, EPS can be used to replace the fatty substances added in bakery products since they produce a structured polysaccharide network, that interacts with the gluten network, mimicking the role of fats maintaining good textural and sensorial properties (Serin and Sayar, 2017; Colla et al., 2018).

Recently, the application of pre-fermented sourdough or fermentation products started with selected technological strains, has led to obtain final products with improved nutritional, technological and sensorial quality and prolonged shelf-life (Katina et al., 2009; Galle et al., 2010; Valerio et al., 2014, 2017; De Bellis et al., 2019; Di Biase et al., 2019). In this perspective, the aim of the current research was to select LAB strains able to produce EPS in liquid sourdoughs (LSs) based on pseudocereal flours and to study the effect of LSs composition (flour type, dough yield, sucrose) on EPS production and protein degradation.

## Materials and Methods

### Raw Materials

The ingredients used in this study were soft wheat flour type 0 (protein, 14,5%; fat, 1%; carbohydrate, 68%) and wheat gluten (protein 75%; fat, 5%; carbohydrate, 15%), both supplied by Valle Fiorita Catering S.r.l. (Ostuni, Italy), organic amaranth flour (protein, 15.5%; fat, 7.1%; carbohydrate, 71.0%) (Sottolestelle srl, San Giovanni Rotondo, Italy), organic quinoa flour (protein, 11.9%; fat, 7.2%; carbohydrate, 56.3%) (Ecor, Verona, Italy) and sucrose (JT Baker, Milan, Italy).

### LAB Strains and Growth Conditions

Twenty-one strains of LAB, belonging to the Culture Collection of the Institute of Sciences of Food Production, National Research Council and deposited in the Belgian Coordinated Collections of Microorganisms (BCCM/LGM, Gent, Belgium) or in the ITEM Collection, were screened for EPS production. *Lactobacillus brevis* 18F, *L. plantarum* ITM21B, *L. plantarum* 19A (Corsetti et al., 2003), *Lactobacillus hilgardii* 51B (Di Cagno et al., 2003), *Lactobacillus sanfranciscensis* C57 (Corsetti et al., 1996) were isolated from sourdough; *Lactobacillus paracasei* IMPC2.1 (LMG P-22043) and *L. paracasei* IMPC4.1 (LMG S-27068) were human isolates (Lavermicocca et al., 2005); *Lactobacillus pentosus* 15BG and *L. pentosus* 14TG were isolated from olive surface (De Bellis et al., 2010); *Leuconostoc citreum* C2-27, *Ln. mesenteroides* C43-18, *Weissella cibaria* strains C21-4, C2-5, C43-11, C3-2, C3-4, C3-19, C4-21, C2-32 and *Weissella confusa* strains C5-4 and C5-7 were isolated from wheat semolina (Valerio et al., 2009).

Strains were cultured in MRS broth (Biolife Italiana S.r.l., Milan, Italy) and incubated at 37°C (*Lactobacillus* spp.) or 30°C (*Weissella* spp.). For long-term storage, stock cultures were prepared by mixing 8 mL of a culture with 2 mL of Bacto glycerol (Difco, Becton Dickinson, Co., Sparks, MD, United States) and freezing 1 mL portions of this mixture at −80°C. Cultures were subcultured twice and incubated at the optimal growth temperature for each strain (30°C *Weissella* spp. and *Leuconostoc citreum* or 37°C *Lactobacillus* spp.) for 24 h before use.

### Screening for EPS Production in Synthetic Media

To identify EPS producers, each bacterial strain was cultivated for 24 h in a modified MRS (mMRS) broth prepared by adding fresh yeast extract (5%, v/v) and 28 mM maltose to the MRS (Biolife Italiana) composition (final pH 5.6) as reported in Di Cagno et al. (2006). Cultures were inoculated on mMRS agar plates enriched with three carbon sources: 292 mM sucrose (mMRS_S), 146 mM glucose (mMRS_G) or 146 mM fructose (mMRS_F). After incubation at 30 or 37°C for 6 days, EPS producing strains were distinguished by visual appearance of mucoid colonies. To quantify the production of EPS, each positive strain was inoculated at 4% (v/v) in mMRS_S broth and cultures were incubated for 24 h at the appropriate temperatures. Liquid mMRS without additional carbohydrates was used as a control. Furthermore, to assess the production of EPS in a shorter time, cultures were also incubated for 15 h at the appropriate temperatures.

Both experiments were carried out twice and analyzed in duplicate (2 × 2).

### Production of Liquid Sourdoughs (LSs)

To study the effect of sucrose on EPS production, preliminary formulations of LSs were obtained by mixing wheat flour (W) with wheat gluten (Gl) or amaranth flour (Am) or quinoa flour (Q) in a 1:1 ratio, in the presence or not of sucrose (S) (3% w/w of LS), giving a dough yield (DY) of 500 (Table 1). The preliminary LSs were inoculated with *W. cibaria* C43-11 (EPS-high producer strain) or *L. plantarum* ITM21B (not producing EPS in mMRS_S). Cells from 24 h cultures in mMRS_S were washed, resuspended in distilled water and inoculated in the flour mixtures at 4% (v/v). Mixtures were incubated at optimal growth temperature for each strain (30°C *W. cibaria*, C43-11 37°C *L. plantarum* ITM21B) for 15 h and the resulting fermentation products were analyzed.

**TABLE 1:** Ingredients of LSs containing wheat flour (W) with gluten (Gl) or amaranth flour (Am) or quinoa flour (Q) (ratio 1:1), in the presence or not of sucrose (S) (3% w/w) (DY 500).

Ingredients	W/Gl+S	W/Gl	W/Am+S	W/Am	W/Q+S	W/Q
Wheat flour type 0	10 g	10 g	10 g	10 g	10 g	10 g
Gluten	10 g	10 g	–	–	–	–
Amaranth flour	–	–	10 g	10 g	–	–
Quinoa flour	–	–	–		10 g	10 g
Water	76 ml	76 ml	76 ml	76 ml	76 ml	76 ml
Sucrose	3 g	–	3 g	–	3 g	–
Culture	4 ml	4 ml	4 ml	4 ml	4 ml	4 ml

A deeper study on the factors affecting EPS synthesis, metabolic activities (organic acids, protein degradation, pH, TTA) and starter viability, was carried out by formulating three different LS types distinguished for the dough yields (250 and 500), pseudocereal/wheat flour ratio (1:1 or 1:0) and sucrose content (3 and 6% LS weight). Mixtures were inoculated with strains *W. cibaria* C43-11 or *L. plantarum* ITM21B and incubated as described above. For each pseudocereal flour, six LSs were obtained as described in Table 2. The same formulations not containing the bacterial inoculum and added with antibiotics (100 mg kg^–1^ chloramphenicol and 50 mg kg^–1^ erythromycin) were used as controls (Co), as reported in Korakli et al. (2002).

**TABLE 2:** Optimization of LS formulations containing pseudocereals (amaranth or quinoa) in combination or not with wheat flour (W) in ratio 1:1, with sucrose 3% or 6% LS weight, at DY 500 or DY250.

Ingredients	LS type^a^					
	DY500_3%S		DY250_3%S		DY250_6%S	
Amaranth/Quinoa flour	10 g	20 g	20 g	40 g	20 g	40 g
Wheat flour type 0	10 g	–	20 g	–	20 g	–
Water	76 ml	76 ml	56 ml	56 ml	56 ml	56 ml
Sucrose	3 g	3 g	3 g	3 g	6 g	6 g
Culture	4 ml	4 ml	4 ml	4 ml	4 ml	4 ml
DY	500	500	250	250	250	250

*^*a*^LS types: wheat flour (W) in combination with amaranth (Am) or quinoa (Q) and sucrose 3% LS weight, DY 500 (W/Am or W/Q_DY500_3%S); amaranth (Am) or quinoa (Q) and sucrose 3% LS weight, DY 500 (Am or Q_DY500_3%S); wheat flour (W) in combination with amaranth (Am) or quinoa (Q) and sucrose 3% LS weight, DY 250 (W/Am or W/Q_DY250_3%S); amaranth (Am) or quinoa (Q) and sucrose 3% LS weight, DY 250 (Am or Q_DY250_3%S); wheat flour (W) in combination with amaranth (Am) or quinoa (Q) and sucrose 6% LS weight, DY 250 (W/Am or W/Q_DY250_6%S); amaranth (Am) or quinoa (Q) and sucrose 6% LS weight, DY 250 (Am or Q_DY250_6%S).*

Experiments were carried out twice and analyzed in duplicate (2 × 2).

### EPS Quantification Method

Samples were prepared for the quantification assay as reported in Di Cagno et al. (2006). Briefly, liquid cultures in mMRS_S were heat treated at 100°C for 15 min to inactivate enzymes and centrifuged (9000 × *g*, 10 min, 4°C). Three volumes of chilled 96–99% (v/v) ethanol were added to the resulting supernatants and the solutions were stored at 4°C overnight. The precipitated EPS were collected by centrifugation (11325 × *g*, 20 min), dissolved in distilled water, dialyzed (12–14 kDa) against distilled water at 4°C for 48 h, lyophilized and rehydrated with distilled water at the initial volume. Whereas, LSs were diluted 1:10 with distilled water, centrifuged (8000 × *g*, 20 min) and the supernatants treated as described above. The concentration of EPS (g/L or g/kg) was determined according to the phenol-sulfuric method (Dubois et al., 1956), using glucose as a standard (LOD 0.078 g/L or g/kg).

### Viscosity Measurement of LSs

The viscosity of LS was measured on 35 mL of each sample using the sine-wave vibro-viscometer SV-10 (A&D Company, Ltd., Tokyo, Japan). Viscosity (expressed as mPa s) was obtained by detecting the driving electric current needed to resonate two sensor plates at a constant frequency of 30 Hz and amplitude of less than 1 mm and at constant room temperature (20 ± 1.0°C).

### Microbiological and Physico-Chemical Analyses

Serial decimal dilutions of mMRS_S 15 h cultures or LSs in sterile NaCl (0.85% w/v) + Tween 80 (0.025%), were prepared, and 100 μl aliquot of each dilution was spread on MRS plates which were incubated for 48 h at the optimal growth temperature for the two selected strains. The total LAB count was expressed as log CFU/ml or CFU/g.

The strains inoculated in LSs were identified as reported in Valerio et al. (2017). Briefly, 20% of total colonies, randomly picked from MRS agar plates containing the two highest dilutions, were isolated and checked for purity. Bacterial DNA was extracted from each colony from overnight cultures grown in MRS broth at 30 or 37°C as previously described (De Bellis et al., 2010). The amplification products were separated by microfluidic electrophoresis using the DNA7500 LabChip kit with the Agilent 2100 Bioanalyzer (Agilent Technologies, Waldbronn, Germany). Chips were prepared according to the manufacturer’s instructions. Data were analyzed using the 2100 Expert software provided by the same company. Genetic identification of strains was based on the comparison of the REP-PCR profile of each isolate with the specific pattern obtained from pure cultures of *L. plantarum* ITM21B and *W. cibaria* C43-11.

The pH of LSs was recorded with a portable pH-meter (type110, Eutech Instruments, Singapore) supplied with Double Pore D electrode (Hamilton, Bonaduz, Switzerland). Total titratable acidity (TTA) was measured according to AOAC Method No. 981.12 (AOAC, 1990) and expressed in mL of 0.1N NaOH required to achieve a pH of 8.3.

### Determination of Organic Acids in LSs

Sample preparation and analysis of lactic, acetic, phenyllactic (PLA) and hydroxy-phenyllactic (OH-PLA) acids was performed as reported in Di Biase et al. (2019). Briefly, 10-g portions of each LS were diluted in sterile tap water (90 mL), homogenized in a Stomacher (Seward, London, United Kingdom) for 2 min, then the suspensions were centrifuged (9072 × *g*, 10 min, 4°C) and the supernatants freeze-dried. The freeze-dried samples were re-suspended in the HPLC mobile phase (0.007 mol/L H_2_SO_4_) (Fluka, Deisenhofen, Germany) and filtered by centrifugation (7000 × *g*, 1 h, 2°C) through a 3000 Da cut-off micro-concentrator (Ultracel-3k, Amicon, Danvers, MA, United States). The fraction containing molecules with molecular weight lower than 3000 Da was analyzed by HPLC (AKTABasic10, P-900 series pump, Amersham Biosciences AB, Uppsala, Sweden; degasser Gastorr BG-12, FLOM Corporation, Tokyo, Japan), using a Rezex ROA organic acid H+ (8%) column (7.80 mm × 300 mm, Phenomenex, Torrance, CA, United States), an injection volume of 10 μL, a 3-channel UV detector (Amersham Biosciences 900) set at 210 (lactic, acetic, PLA acids) and 220 nm (OH-PLA). The mobile phase was pumped at a flow rate of 0.7 mL/min through the column heated to 65°C. Quantification of the organic acids was performed by integrating calibration curves obtained from the relevant standards. Limit of detection (LOD) and limit of quantification (LOQ) were calculated considering a signal-to-noise ratio (S/N) of 3 and 6, respectively. LOD values were the following: lactic acid, 0.263 mmol/kg; acetic acid, 0.279 mmol/kg; PLA, 1.08 μmol/kg; OH-PLA, 0.774 μmol/kg. LOQ values corresponded to 2 × LOD. Final concentration of each organic acid in LSs was calculated considering concentration and/or dilution factors and expressed as mmol/kg or μmol/kg of product.

### Protein Characterization of Flour Samples and LSs

Total proteins were extracted from flours and LSs (before and after fermentation) under reducing conditions. Briefly, 40 mg of flour – or an equivalent amount of LSs weighed on the basis of their flour content – were mixed with 200 μL of extraction solution containing 5% mercaptoethanol and 2% SDS (Džunková et al., 2011) for 3 h at 4°C. Afterward insoluble material was removed by centrifugation (12000 × *g* for 15 min) and supernatants, after heating at 100°C for 2 min, were stored at −20°C and used for electrophoresis. Protein concentration was determined by the Bradford method (Bradford, 1976) using the Bio-Rad dye reagent (Bio-Rad Laboratories, Hercules, CA, United States) with bovine serum albumin as the standard and expressed as mg of protein per gram of flour. The total protein degradation (TPD) during fermentation was expressed as a percentage and quantified using the following equation:

(1) $$\%TPD=100-\left[\left(\frac{T\,P_{f}}{T\,P_{i}}\right)\times 100\right]$$

Where TP_*f*_ represents the total protein content after fermentation and TP_*i*_ is the initial total protein content (before fermentation).

Total protein extracts were analyzed by the Lab-on-a-Chip (LoaC) capillary electrophoresis using the Protein 230 Lab Chip kit (Agilent Technologies, Waldbronn, Germany) with a molecular weight range of 14–230 kDa. Sample preparation and chip loading was performed according to manufacturer’s instructions and data evaluation was carried out by the dedicated 2100 Expert software that aligns sample proteins to a molecular weight ladder using internal standards. Results were displayed for each sample as peaks (electropherogram), as bands (gel-like image) and in a tabular format that reports, for each protein peak, molecular weight (Mw), time-corrected peak area (TCA), relative concentration (RC) based on a one-point calibration to the upper marker (60 ng/mL) and protein percentage (%) calculated on total peak areas in the sample. Manual integration of peaks was performed after each run and peaks with RC <20 ng/μl were excluded from analysis as their significance is low considering the detection limit of the method. All experiments were performed twice (*n* = 2).

### Statistical Analysis

All data are presented as mean values ± standard error of the mean. To evaluate the effect of formulations characterized by different DY, sucrose content, flour type and ratio and bacterial starter (ITM21B, C43-11, Co) on the physico-chemical parameters (EPS, %TPD, viscosity, pH, TTA, organic acids lactic, acetic, PLA, and OH-PLA) of LSs, data were analyzed by factorial ANOVA followed by the Tukey HSD test. Results were considered as statistically significant when the *p*-value was less than 0.05. All statistical analysis were performed by Statistica 13 software (Dell Inc., 2015). Data were analyzed by principal component analysis (PCA) to investigate the correlation between the physico-chemical parameters. Multivariate analysis was performed by the Unscrambler (version 10.1, CAMO, Oslo, Norway).

## Results

### Screening for EPS Production

Twenty-one strains were tested on mMRS agar plates containing different carbon sources (glucose, fructose, or sucrose) and the *W. cibaria*, *W. confusa*, and *Leuconostoc* spp. strains resulted to be the most active EPS producers, but only in the presence of sucrose (Supplementary Table S1). In particular, the 10 *Weissella* spp. strains producing EPS in the presence of sucrose as carbon source and a not-producing strain (*L. plantarum* ITM21B), were grown for 24 h in liquid medium (mMRS_S) containing sucrose 292 mM (10% w/v): the higher amount of EPS (18.56 ± 2.64 g/L) was registered for strain C43-11 while for ITM21B the EPS production was not detected (Table 3).

**TABLE 3:** Production of EPS in mMRS_S after 24 h growth by selected producer strains (*Weissella cibaria* and *Weissella confusa*) in comparison to the strain *L. plantarum* ITM21B not producing EPS in agar media.

Species	Strain	EPS g/L ± SE
*W. cibaria*	C21-4	13.82 ± 1.88ac
	C3-2	13.42 ± 3.45ac
	C3-4	6.21 ± 2.12ab
	C43-11	18.56 ± 2.64c
	C3-19	14.82 ± 0.66ac
	C4-21	8.20 ± 3.29abc
	C2-32	0.51 ± 0.64b
	C2-5	8.02 ± 3.36abc
*W. confusa*	C5-7	2.08 ± 0.03ab
	C5-4	1.08 ± 0.39ab
*L. plantarum*	ITM21B	<LOQ

*Values (mean ± standard error of the mean) with different lower-case letters are significantly different (*p* < 0.05). LOD: 0.078 g/L.*

In order to produce a LS in a shorter time, the two selected strains C43-11 and ITM21B were grown in mMRS_S for 15 h, an incubation time generally used to produce fermentation products suitable for bread-making (Valerio et al., 2017; Di Biase et al., 2019). Results indicated that already after 15h of incubation, strain C43-11 was able to produce EPS (16.1 ± 0.1 g/L) while ITM21B grew better (9.54 ± 0.007 vs. 8.80 ± 0.01 log CFU/ml, *p* < 0.05) determining lower pH values (3.96 ± 0.002 vs. 4.33 ± 0.01, *p* < 0.05) but comparable TTA values (21.5 ± 0.61 for C43-11 and 20.2 ± 3.80 for ITM21B, *p* > 0.05). Therefore, even if a lower EPS production was registered after 15 h respect to 24 h growth, this fermentation time was used in the further experiments using wheat and pseudocereal flours as carbon source in the presence or not of sucrose.

### Effect of Sucrose on EPS Production in Preliminary Liquid Sourdough Formulations

**FIGURE 1:**
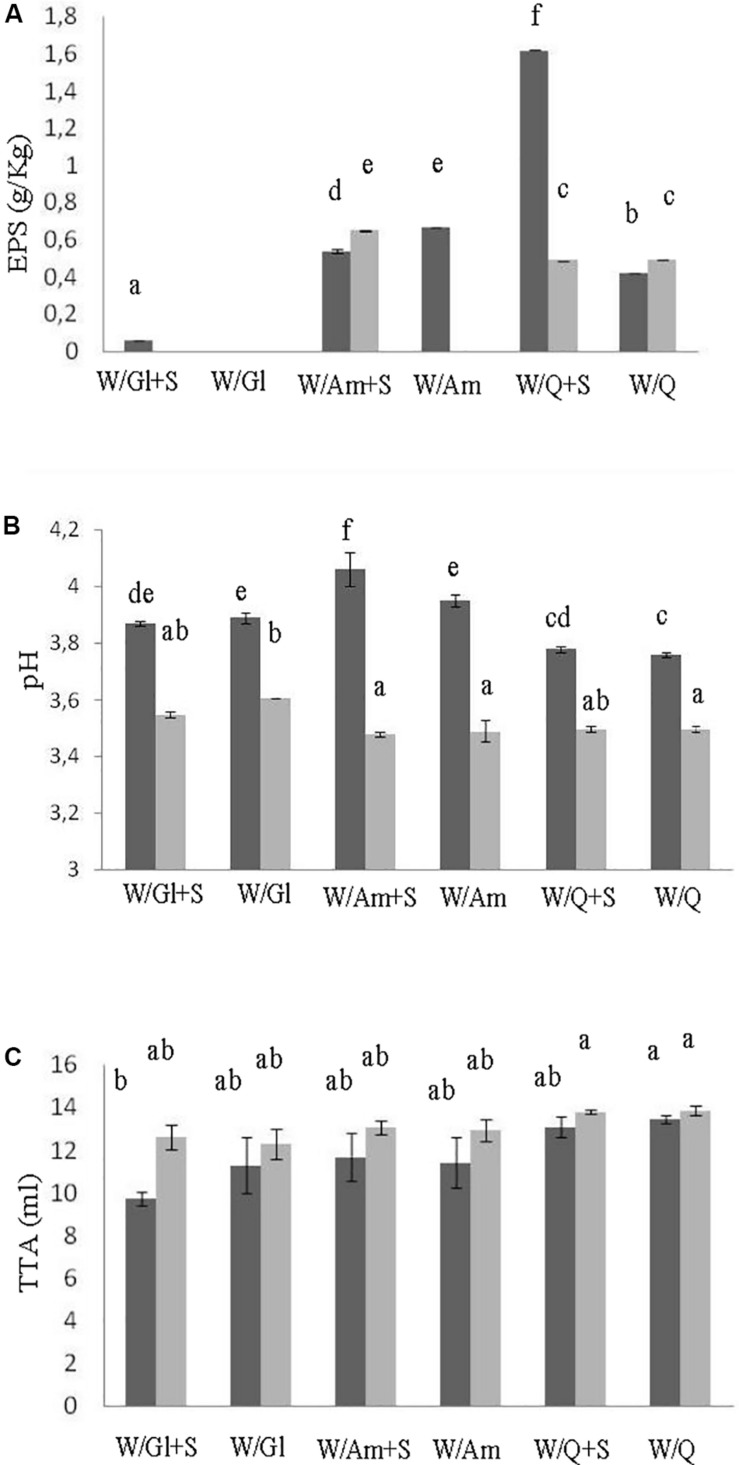
Effect of different flour combinations on EPS content **(A)**, pH **(B),** and TTA **(C)** of liquid sourdoughs (LSs) obtained by the selected high EPS producer *W. cibaria* strain C43-11 (dark gray) and the low EPS producer strain *L. plantarum* ITM21B (light gray) after 15 h fermentation. Ingredients: wheat flour (W) with gluten (Gl) or amaranth flour (Am) or quinoa flour (Q) (ratio 1:1), in the presence or not of sucrose (S) (3% on LS weight). The data are represented as mean ± standard error. Different letters above the columns indicate significant difference among the means of EPS content **(A)**, pH **(B)**, and TTA **(C)** by Tukey HSD’s test (*p* < 0.05).

**FIGURE 2:**
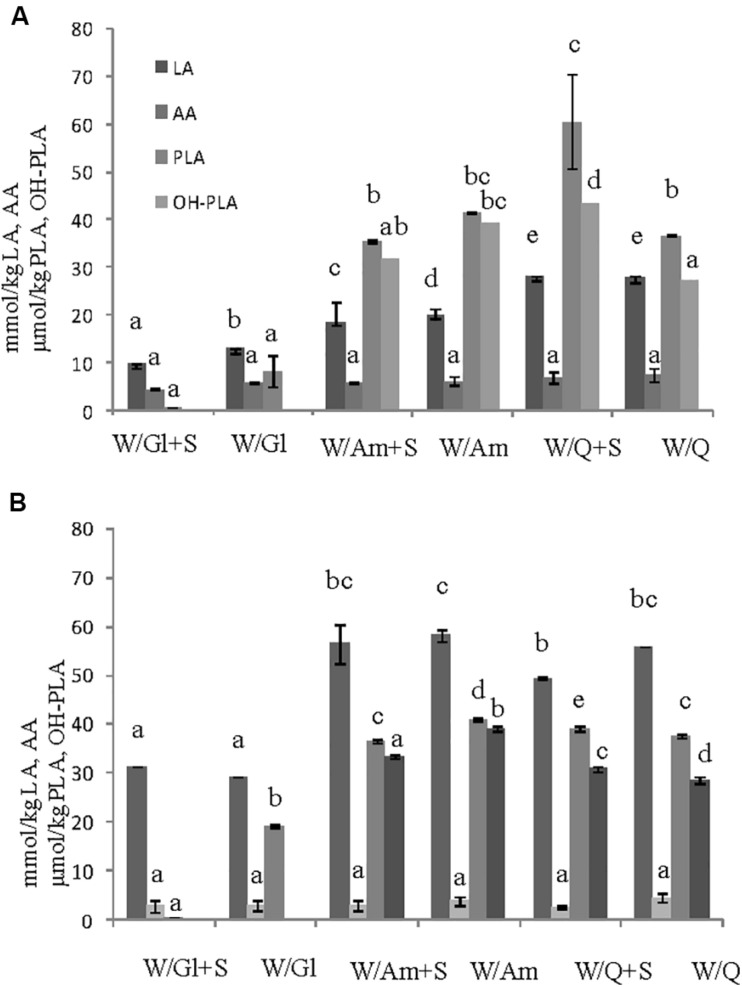
Effect of different flours on the organic acid content (lactic, LA, acetic, AA, phenyllactic, PLA, OH-phenyllactic, OH-PLA) registered in the preliminary LSs obtained by the **(A)** *W. cibaria* C43-11 and the **(B)** *L. plantarum* ITM21B after 15 h fermentation. Ingredients: wheat flour (W) with gluten (Gl) or amaranth flour (Am) or quinoa flour (Q) (ratio 1:1), in the presence or not of sucrose (S) (3% w/w). The data are represented as mean ± standard error. Different letters above the columns indicate significant difference among the means of each organic acid by Tukey HSD’s test (*p* < 0.05).

In order to assess the contribute of sucrose on EPS production three LS formulations at DY 500 based on wheat flour with pseudocereals or wheat gluten as sources of carbohydrates, in the presence or not of sucrose (3% LS weight), were prepared. The flour mixtures were inoculated with *W. cibaria* C43-11 or *L. plantarum* ITM21B, a strain not producing EPS in the above tested conditions, and fermented for 15 h at 30 or 37°C. LSs were characterized for the pH, TTA, LAB count, EPS content and acid production. As shown in Figure 1A, the presence of pseudocereals favored EPS production by strain ITM21B, even if at lower concentration respect to strain C43-11. The higher EPS production was registered in C43-11 W/Q+S LS even if at levels considerably lower (1.62 ± 0.01 g/kg, *p* < 0.05) than that observed in mMRS_S. The addition of sucrose at 3% (w/w LS weight, corresponding to the 15% on flour weight) significantly improved the EPS production by C43-11 strain only in LS containing quinoa/wheat flours. The strain count ranged between 7.0 ± 0.02 and 9.0 ± 0.02 log CFU/g for strain C43-11 and between 8.5 ± 0.01 and 10.2 ± 0.01 log CFU/g for strain ITM21B (*p* < 0.05). For each strain, a higher bacterial count was registered in formulations containing quinoa or amaranth (data not shown). Lower (*p* < 0.05) pH values and slightly (*p* > 0.05) higher acidity (TTA) and lactic acid were observed for all LSs fermented by ITM21B strain (Figures 1B,C). All LSs formulations including gluten, showed the lowest content of organic acids (Figure 2), highlighting a scarce proteolysis. The production of lactic acid, PLA and OH-PLA was related to the flour type and to the presence of pseudocereals as a high production of these metabolites was observed in all LSs containing quinoa or amaranth. In particular, the highest content of PLA and OH-PLA (60.55 ± 9.92 and 43.6 ± 6.3 μmol/kg, respectively) was observed in W/Q_S LS fermented by strain C43-11 (Figure 2A). These metabolites are known for their antimicrobial properties and for their contribution to the taste of salt-reduced bread (Valerio et al., 2008, 2017). Strain ITM21B produced lactic acid at a significantly (*p* < 0.05) higher level, mainly in the presence of pseudocereals, respect to strain C43-11 while the remaining acids were comparable (Figure 2B). Notably, except for EPS production, all chemical and microbiological results in LSs were not affected by the presence of sucrose, thus suggesting the contribution of proteins and aminoacids contained in pseudocereals on the modifications observed for pH, TTA, and organic acids.

### Effect of LS Formulation on EPS Production, Microbiological and Physico-Chemical Parameters, and Protein Profile

**FIGURE 3:**
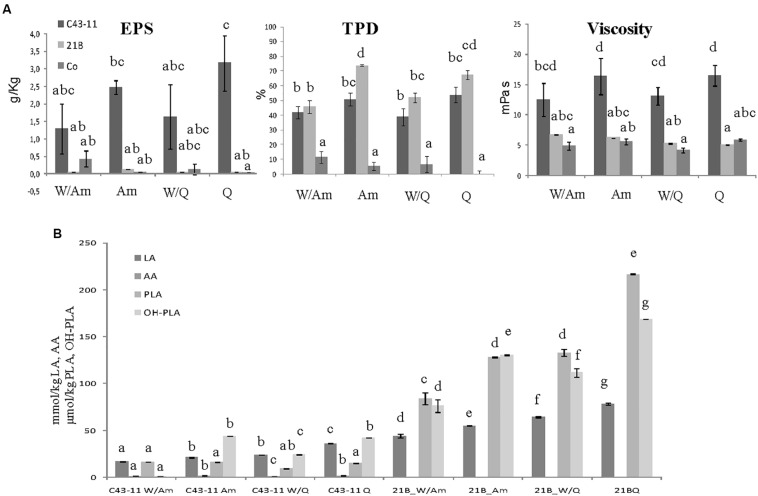
Effect of pseudocereal flours in combination or not with wheat flour on **(A)** EPS content, viscosity, total protein degradation (TPD%) and **(B)** organic acid content in LSs obtained by *W. cibaria* C43-11, or *L. plantarum* ITM21B and in control LS after 15 h fermentation. Ingredients: wheat flour (W) in combination (ratio 1:1) or not with amaranth flour (Am) or quinoa flour (Q), in the presence of sucrose (S) (3% LS weight) at DY 500. The data are represented as mean ± standard error. Different letters above the columns indicate significant difference among the means of EPS content, pH, and TTA **(A)** and among each organic acid **(B)** by Tukey HSD’s test (*p* < 0.05).

**FIGURE 4:**
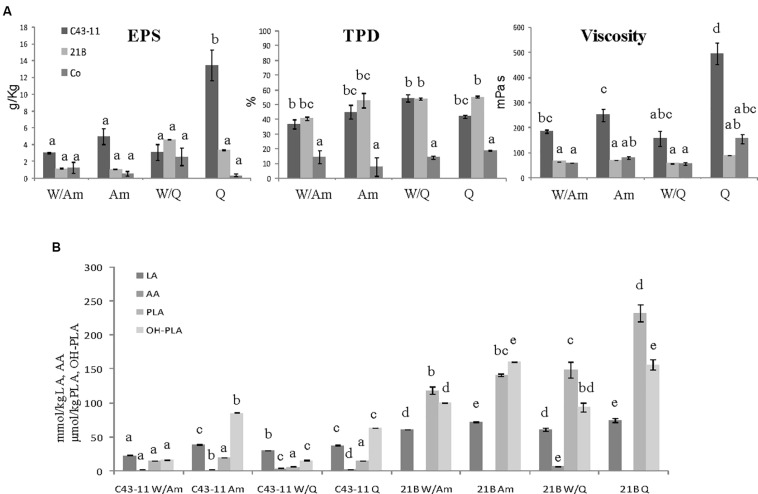
Effect of pseudocereal flours in combination or not with wheat flour on **(A)** EPS content, viscosity, total protein degradation (TPD%) and **(B)** organic acid content in LSs obtained by *W. cibaria* C43-11, or *L. plantarum* ITM21B and in control LS after 15 h fermentation. Ingredients: wheat flour (W) in combination (ratio 1:1) or not with amaranth flour (Am) or quinoa flour (Q), in the presence of sucrose (S) (3% LS weight) at DY 250. The data are represented as mean ± standard error. Different letters above the columns indicate significant difference among the means of EPS content, pH, and TTA **(A)** and among each organic acid **(B)** by Tukey HSD’s test (*p* < 0.05).

**FIGURE 5:**
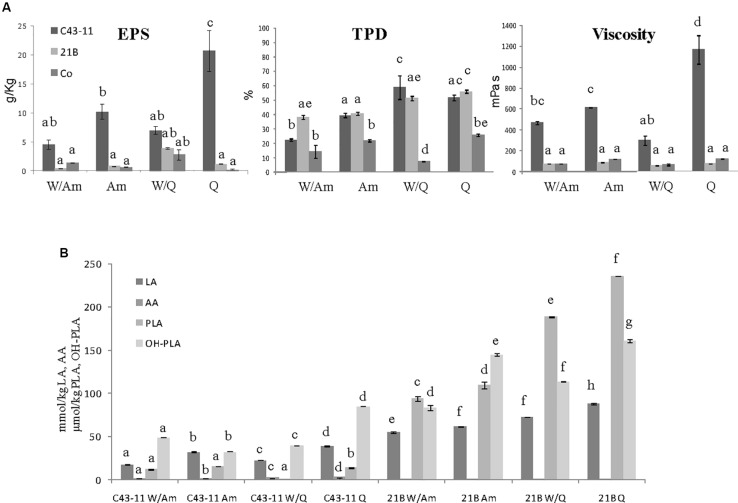
Effect of pseudocereal flours in combination or not with wheat flour on **(A)** EPS content, viscosity, total protein degradation (TPD%) and **(B)** organic acid content in LSs obtained by *W. cibaria* C43-11, or *L. plantarum* ITM21B and in control LS after 15 h fermentation. Ingredients: wheat flour (W) in combination (ratio 1:1) or not with amaranth flour (Am) or quinoa flour (Q), in the presence of sucrose (S) (6% LS weight) at DY 250. Data are represented as mean ± standard error. Different letters above the columns indicate significant difference among the means of EPS content, pH, and TTA (A) and among each organic acid (B) by Tukey HSD’s test (*p* < 0.05).

To study the influence of the starter strain (*W. cibaria* C43-11 or *L. plantarum* ITM21B), DY (500 or 250), flour composition and ratio (W/Am, Am, W/Q, Q) and sugar content (3 or 6% w/w of LS) on EPS production, metabolic activities (organic acids, total protein degradation, pH, TTA) and starter viability, three LS types were formulated (Figures 3–5). They were mainly distinguished by DY and sugar content (DY500, 3% sucrose; DY250, 3% sucrose; DY250, 6% sucrose).

Since preliminary results (Figures 1, 2) suggested the use of pseudocereals to increase the EPS production, LSs at DY 500 and with sucrose 3% containing only the pseudocereal, were produced. As shown in Figure 3A the flour type and its proportion respect to wheat flour had a significant effect on the EPS production: *W. cibaria* C43-11 confirmed the production of EPS, in all DY500 LSs, particularly in that containing only quinoa (3.17 ± 0.78 g/kg), even if at levels considerably lower (*p* < 0.05) than those observed in mMRS_S (16.1 ± 0.1 g/L). No significant differences (*p* > 0.05) in the viscosity values among the DY500 3%_S LSs within each strain were observed, but C43-11 based LSs showed the higher values (*p* < 0.05) respect to strain ITM21B and Co. A significant increase of the bacterial populations (from a median value of 7.11 to 9.18 log CFU/g for C43-11 and from 7.88 to 9.54 log CFU/g for strain ITM21B) was registered in all inoculated LSs. Values of pH in LS fermented by strain C43-11 were quite similar (range: 3.95÷4.04, *p* > 0.05), while a significant modification was observed in LSs fermented by strain ITM21B changing the flour type: the lowest value was observed in W/Am LS (3.25 ± 0.02), followed by Am LS (3.40 ± 0.002) and quinoa based LSs (3.74 ± 0.002). Whereas, the pH of Co LSs remained unvaried at values of 6.30÷6.42. However, the TTA values were always higher in ITM21B LSs containing quinoa flour (Table 4). The production of organic acids was considerably higher in ITM21B based LSs, except for acetic acid which was produced at a low extent only by the strain C43-11 (Figure 3B). Consequently the total protein degradation (% TPD) mainly occurred in the presence of strain ITM21B, particularly in quinoa and amaranth flours, in which a 67.3 ± 3.04 and 73.7 ± 0.7% reduction was observed.

**TABLE 4:** Values of TTA in optimized LSs, at DY 250 or 500, fermented by *W. cibaria* C43-11 or *L. plantarum* ITM21B in comparison to the uninoculated control (Co), after 15 h incubation.

Flour	Co	C43-11	ITM21B
**DY 500, 3% Sucrose**			
W/Am	1.40 ± 0.04a	6.05 ± 0.02d	9.90 ± 0.16e
W/Q	1.73 ± 0.05a	9.25 ± 0.10e	14.00 ± 0.20ghi
Am	2.15 ± 0.06a	10.25 ± 0.06ef	14.75 ± 0.10ghi
Q	2.78 ± 0.19abc	13.15 ± 0.14gh	18.00 ± 0.61jkl
**DY 250, 3% Sucrose**			
W/Am	2.78 ± 0.09abc	10.25 ± 0.02ef	15.75 ± 0.10hij
W/Q	5.08 ± 1.22bcd	14.85 ± 0.14ghi	23.40 ± 0.16no
Am	3.98 ± 0.21abcd	18.00 ± 0.04jkl	22.00 ± 0.41mn
Q	5.43 ± 0.38cd	20.25 ± 0.92lm	26.75 ± 0.92p
**DY 250, 6% Sucrose**			
W/Am	2.60 ± 0.04ab	10.35 ± 0.10ef	14.40 ± 0.12ghi
W/Q	5.28 ± 1.64bcd	12.75 ± 0.31fg	20.75 ± 0.92lmn
Am	3.55 ± 0.06abcd	16.25 ± 0.18ijk	19.55 ± 0.02lm
Q	5.40 ± 0.32cd	18.55 ± 0.18kl	26.15 ± 1.08op

*Ingredients: wheat flour (W) with amaranth flour (Am) or quinoa flour (Q) (ratio 1:1) in the presence of sucrose (S) (3% or 6% LS weight). Values (mean ± standard error of the mean) with different lower case letters are significantly different among all (*p* < 0.05).*

When the DY was lowered to 250, maintaining the sucrose level at 3% LS weight, the most significant changes were observed in C43-11 Q LS which showed the highest EPS production (13.43 ± 1.83 g/kg) and relevant viscosity (495.25 ± 43 mPa s) (Figure 4A). In addition, also strain ITM21B resulted able to produce an appreciable EPS amount (4.61 ± 0.01 g/kg LS corresponding to about 11 g/kg of flour) in W/Q LS, with a concomitant protein degradation of more than 50%, even if the viscosity remained at a low level (56.9 ± 0.1 mPa s). In the overall, TTA values significantly (*p* < 0.05) increased in all samples (Table 5) even if for pH values, range: 4.05÷4.29 for C43-11, 3.48÷3.97 for ITM21B, 5.88÷6.31 for Co and acetic acid content (Figure 4B), only a slight increase was observed. Strain ITM21B determined a significantly lower (*p* < 0.05) pH value in W/Am LS (3.48 ± 0.002) respect to the other formulations (Am 3.68 ± 0.01, W/Q 3.87 ± 0.002 and Q 3.97 ± 0.004). The same behavior was observed for strain C43-11 since the lower (*p* < 0.05) pH value was registered in W/Am LS (4.05 ± 0.01) followed by Am (4.14 ± 0.002), W/Q (4.2 ± 0.004), and Q (4.29 ± 0.01). In LSs at DY 250, the content of flour, and consequently of proteins, was twofold higher than LS DY 500 (Supplementary Table S2) but the protein degradation (% TDP) remained almost unvaried (*p* > 0.05) indicating that a lower DY favors proteolysis (Figure 4A). Interestingly, in the presence of strain C43-11, proteins were hydrolyzed at the same extent of LS fermented by *L. plantarum* (*p* > 0.05) and percentage values were slightly higher in Am (44.9 ± 4.6%) and W/Q (54.4 ± 2.4%) LSs (Figure 4A).

**TABLE 5:** Distribution of proteins (expressed as protein percentage on total peak areas) in Mw areas (A1, A2, A3) and molecular weight ranges of proteins for each LS type, as a result of LoaC capillary electrophoretic analysis on Protein 230 Labchip, before and after fermentation with *Weissella cibaria* C43-11 or *Lactobacillus plantarum* ITM21B and in comparison to the uninoculated control (Co).

	Am				W/Am				Q				W/Q			
	
	LS DY500_3%S															
Mw area (kDa)	T0	Co	ITM21B	C43-11	T0	Co	ITM21B	C43-11	T0	Co	ITM21B	C43-11	T0	Co	ITM21B	C43-11
A1 (14–30)	40.9	56.7	96.9	88.7	25.1	27.8	40.7	40.2	20.1	41.8	100	94.3	20.4	26.2	78.9	75.4
A2:																
I (31–42)	47.8	40.1	3.1	11.3	31.4	42.9	40.7	41.7	22.1	40.6	0	5.7	18.3	21.9	4.6	13.2
II (43–55)	8.5	1.7	0	0	25.0	15.7	15.6	12.8	40.0	8.2	0	0	43.7	28.7	3.7	4.0
III (56–79)	2.2	1.5	0	0	13.6	11.0	3.0	5.3	13.7	6.9	0	0	13.6	13.7	10.0	7.4
A3 (>80 kDa)	0.6	0	0	0	4.9	2.6	0	0	4.1	2.5	0	0	4.0	9.5	2.8	0
Mw range (kDa)	14–94	14–79	14–35	14–35	14–222	14–225	14–57	14–57	14–117	14–105	14–30	14–32	14–225	14–225	14–103	14–57

**LS DY250_3%S**																
A1 (14–30)	43.8	55.8	89.4	88.6	24.5	24.4	73.0	37.7	24.1	37.4	93.5	70.1	14.2	20.7	95.3	55.1
A2:																
I (31–42)	46.0	40.8	10.6	11.4	33.0	36.1	15.5	33.5	27.3	39.8	6.5	27.0	4.0	17.8	4.7	21.1
II (43–55)	7.6	1.3	0	0	25.1	29.8	10.7	16.4	32.3	10.7	0	0	48.4	34.0	0	16.5
III (56–79)	2.0	2.1	0	0	13.6	5.7	1.9	6.9	11.4	9.1	0	0.9	25.5	17.0	0	7.3
A3 (>80 kDa)	0.6	0	0	0	3.8	4.0	0	5.5	4.9	3.0	0	2.0	7.9	10.5	0	0
Mw range (kDa)	14–96	14–79	14–32	14–35	14–226	14–225	15–57	14–220	14–117	14–104	14–36	14–97	14–214	14–225	14–39	14–60

**LS DY250_6%S**																
A1 (14–30)	46.2	62.4	94.2	74.2	34.8	27.3	68.8	53.2	20.4	40.3	93.8	64.1	16.0	21.0	100	53.4
A2:																
I (31–42)	42.3	33.8	5.8	25.8	21.6	33.8	16.9	26.1	21.1	41.7	6.2	33.9	13.2	18.7	0	28.0
II (43–55)	8.8	1.7	0	0	21.2	24.9	10.8	14.3	42.1	8.1	0	0	32.0	37.8	0	12.0
III (56–79)	2.0	2.2	0	0	13.9	10.0	0	0.5	13.9	7.1	0	2.0	32.1	16.2	0	5.7
A3 (>80 kDa)	0.7	0	0	0	8.5	4.0	3.5	5.9	2.5	2.8	0	0	6.7	6.3	0	0.9
Mw range (kDa)	14–96	14–79	14–43	14–41	14–217	14–219	14–123	14–130	14–117	14–105	14–37	14–57	14–230	14–220	14–21	14–88

The further increase of sucrose concentration to 6% (w/w of LS), allowed to considerably enhance the EPS production and viscosity, mainly in Q and Am LSs fermented by C43-11 strain (Figure 5A). In particular, the EPS production by strain C43-11 reached the value of 20.79 ± 3.55 g/kg LS and a relevant viscosity of 1168.2 ± 139.4 mPa s demonstrating the special attribute of this strain in producing these molecules, while the EPS production remained unvaried for strain ITM21B. Regarding the other parameters, only slight changes were observed (Figure 5A). The pH value ranged between 4.07 and 4.26 for C43-11, 3.49 and 3.93 for ITM21B and 5.98 and 6.26 for Co and were similar (*p* > 0.05) to values of LSs at DY250 with sucrose 3%. In addition TTA values were similar or a little lower respect to those registered in LSs with DY 250 and sucrose 3% (Figure 5B) and the organic acid production did not significantly change. Whereas, a slightly higher protein degradation was observed in quinoa based LSs respect to the Am based LSs.

**FIGURE 6:**
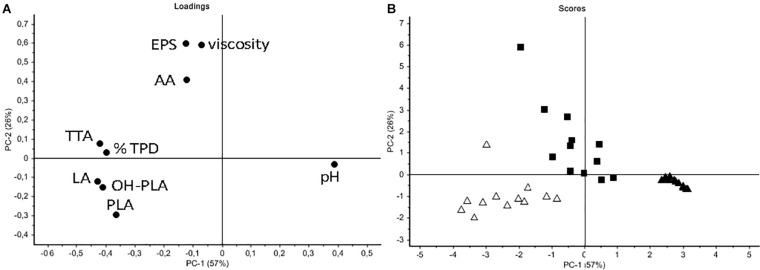
Principal component analysis (PCA) of all data (from the optimized LSs formulated as in Table 2). **(A)** Loading plot indicating the correlation between variables (pH; TTA; % total protein degradation, TPD; organic acids – lactic, LA; acetic, AA; phenyllactic, PLA; hydroxy-phenyllactic OH-PLA), EPS content and viscosity. **(B)** Score plot indicating sample distribution basing on the inoculated strains (black square C43-11; open triangle ITM21B; black triangle Co).

In order to better discriminate LS samples, a PCA was performed on all data (pH, TTA, EPS, viscosity, organic acids, protein degradation) (Figure 6). As shown in the loading plot (Figure 6A), a high correlation between EPS content and viscosity of LSs was registered, indicating that this parameter can be useful to follow the carbohydrate polymer production. A good discrimination among samples mainly based on the strain used to ferment flours, was observed (Figure 6B). Control LSs located on the right of the score plot while the C43-11 LSs were sufficiently discriminated by the ITM21B LSs. Even if at very low level, acetic acid was mainly produced by strain C43-11 while the other acidic molecules were found in LSs fermented by strain ITM21B. In particular, C43-11 LSs were mainly characterized by higher EPS content and viscosity, while ITM21B LSs contained higher amounts of acids and higher TTA and % TPD. Results clearly indicated that an appreciable EPS production (4.61 ± 0.01 g/kg LS corresponding to about 11 g/kg of flour) can be obtained also for the low producer strain ITM21B in W/Q LS at DY 250 and sucrose 3%, with a concomitant protein degradation of more than 50%.

### Proteolysis in LSs

**FIGURE 7:**
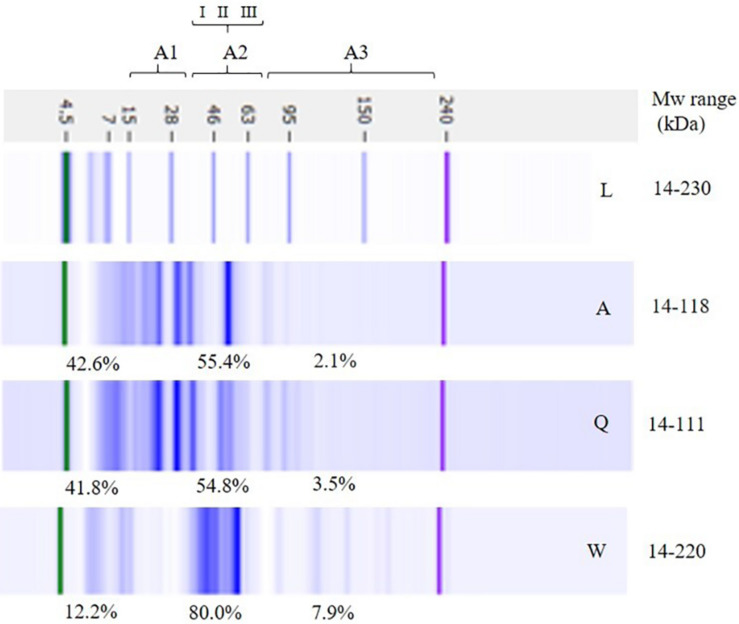
Electrophoretic analysis (LoaC) of total proteins from amaranth (Am), quinoa (Q), and wheat (W) flours and molecular weight marker (L) shown as gel-like images on Protein230 LabChip. Brackets indicate molecular weight areas: A1 (14–30 kDa), A2 (I, 31–42 kDa; II, 43–55 kDa; III, 56–79 kDa), and A3 (>70 kDa). Molecular weight (Mw) and percentage of proteins (%) in each area are the average of two separate experiments (*n* = 2).

Total proteins of flours and modifications in LSs after fermentation were deeply investigated using LoaC capillary electrophoresis. Figure 7 shows a representative gel-like image of the protein profile of flours highlighting the presence of about 15 protein bands ranging from 14 to 220 kDa in wheat flour and between 14 and 118 kDa in amaranth and quinoa flours. Banding patterns were characterized by common proteins and additional specific bands, i.e., 70, 143, and 180 kDa in wheat flour and 20, 31, and 78 kDa in amaranth and quinoa flours.

According to Mw, three different areas were considered in the electropherograms: A1 (14–30 kDa), A2 (31–79 kDa), and A3 (80–230 kDa) and percentages of peaks for each Mw area were gained and are summarized in Figure 7. The major differences were observed in A2, therefore that area was further separated in three sectors (I, II, III). In wheat flour, A2 accounted for the major protein content (about 80%) and it was characterized by main protein bands in the 43–55 kDa range.

In amaranth and quinoa flours, peaks were mainly detected in A1 (about 42%) and A2 (about 55%). In amaranth flour, the presence of double peaks at 16–20 and 31–37 kDa, and two peaks at 41 and 55 kDa, indicated the presence of amaranthin, while the major peaks detected in quinoa flour, i.e., 30–38 and 19–24 kDa are likely to represent the principal protein of quinoa, the chenopodina (Hager et al., 2012). In all flours, Mw A3 contained a low percentage of peaks, i.e., 7.9, 3.5, and 2.1% in wheat, quinoa, and amaranth flours, respectively.

**FIGURE 8:**
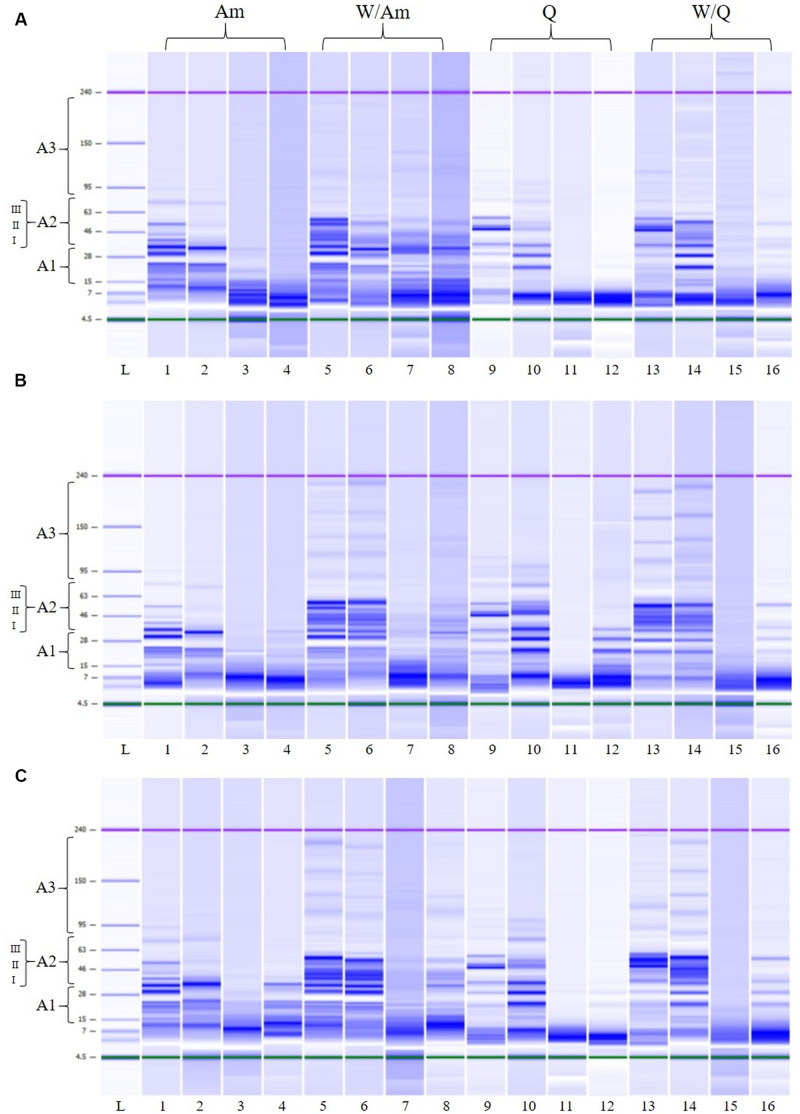
Electrophoretic analysis (LoaC) of total proteins from amaranth (Am), wheat/amaranth (W/Am), quinoa (Q), and wheat/quinoa/ (W/Q) liquid sourdoughs (LSs) with DY500_3%S **(A)**, DY250_3%S **(B)**, and DY250_6%S **(C)** shown as gel-like images on Protein230 LabChip; Molecular weight marker (L); LS uninoculated control T0 (lanes 1, 5, 9, 13); LS uninoculated control T15 (lanes 2, 6, 10, 14); *L. plantarum* ITM21B (lanes 3, 7, 11, 15); *W. confusa* C43-11 (lanes 4, 8, 12, 16). Numbered brackets indicate molecular weight areas: A1 (14–30 kDa), A2 (I, 31–42 kDa; II, 43–55 kDa; III, 56–79 kDa), A3 (>70 kDa).

In order to monitor the flour proteolysis in LSs samples, LoaC analysis was performed before and after fermentation (Figure 8 and Table 5). In detail, at the beginning of fermentation (T0) in all LSs, banding patterns of about 18 protein bands in the 14-225 kDa range were observed for W/Am and W/Q LSs, respectively while about 14 protein bands were found in the 14–96 and 14–117 a kDa range for Am and Q LSs, respectively. As visualized in Figure 8, after 15 h of fermentation the pattern profile of all inoculated LSs considerably changed since high molecular weight proteins were degraded and a concomitant increase of smaller proteins was observed. In uninoculated LSs controls no change in the protein pattern respect to the beginning of fermentation was observed.

Table 5 and Figure 8 summarize the protein changes in the Mw areas for the three LS types.

In inoculated LSs at DY 500, at the beginning of fermentation, proteins were distributed in the three Mw areas (Figure 8A and Table 5) as observed in the relevant flours. After fermentation, in Q and Am LSs, proteins were mainly found in A1 and at a lesser extent in A2, particularly in the 31–42 kDa range, indicating the formation of small proteins due to proteolysis. In the presence of wheat, proteins were observed in the whole A2 region (31–79 kDa) at different percentages depending on the pseudocereal.

Lowering the DY to 250 (3 or 6% sucrose), no significant changes in the protein distribution were observed except for the A2 region of W/Am and W/Q LSs fermented by ITM21B (Figures 8B,C). In particular, proteins in the 43–55 kDa region containing the gliadin and glutenin fractions of wheat, were reduced or completely degraded in W/Am and W/Q LSs, respectively.

Finally, in all LSs, as already observed in their relevant flours, a low % of peaks was found in the A3 region and no significant changes occurred during fermentation.

## Discussion

The use of pseudocereals was considered since their high nutritional value and applicability in gluten-free bread. Among different sources of carbohydrates, the pseudocereals quinoa and amaranth can provide a high content of starch and essential nutrients (Comai et al., 2011; Singh and Singh, 2011), but they don’t produce a viscoelastic dough as for wheat containing gluten. EPS producer LAB strains can successfully pilot the sourdough fermentation favoring the proteolysis and production of microbial carbohydrate polymers. EPS, acting as textural improvers similarly to plant hydrocolloids, remedy the technological drawbacks due to the lack of gluten occurring in gluten-free bakery products or with reduced fat content. Furthermore, EPS production in sourdough meets producer and consumer requirements for “clean label” products.

Several studies on EPS production report the *Weissella* spp. and *Leuconostoc* spp. as high producer genera (Byme, 2001; Di Cagno et al., 2006; Katina et al., 2009; Galle et al., 2010; Wolter et al., 2014). In the current study, all *Weissella* species tested resulted to produce these metabolites in mMRS containing sucrose 10%. In particular, the *W. cibaria* C43-11 was selected as a high producer. Similarly, Zannini et al. (2013) found a *W. cibaria* strain MG1 producing high amount of EPS in similar conditions. As previously reported, the EPS production is highly related to the sucrose concentration, the incubation time and the carbon source (Katina et al., 2009; Rühmkorf et al., 2012; Yu et al., 2018).

With the aim of producing LSs enriched with *in situ* produced EPS, the high producer strain *W. cibaria* C43-11 and the low producer sourdough strain *L. plantarum* ITM21B, were used to ferment wheat flour and/or pseudocereals (quinoa and amaranth). Interestingly, the *L. plantarum* strain, not producing EPS in mMRS_S, resulted to be able to synthesize these molecules, even if at low levels, in the presence of the pseudocereal flours and this ability was strongly affected by the DY and flour type. This effect was amplified for strain C43-11 whose EPS production was optimal in quinoa based LS at DY 250 and containing sucrose 6%. In fact, in the optimal conditions strain C43-11 produced about 20.79 g/kg sourdough, a concentration considerably higher than that produced by *W. cibaria* strains in sourdoughs from different flours (Galle et al., 2012a, b; Wolter et al., 2014; Hu and Gänzle, 2018). Our results confirmed the suitability of quinoa as a substrate for EPS production as previously observed by Rühmkorf et al. (2012). Authors studied the effect of different flours, DY and sucrose addition on EPS yield after fermentation with three *Lactobacillus* species. They found the highest EPS production (20.63 g/kg flour) in quinoa sourdough (DY 250, sucrose 7.5% flour weight, corresponding to our 3% sucrose/LS weight at DY250) after 24 h fermentation. When the sucrose was increased to the 15% flour weight (corresponding to our 6% LS weight at DY250) strain *L. reuteri* TMW 1.106 produced about 25 g/kg flour in 24 h. In our study, the highest EPS yield (20.79 ± 0.2 g/kg LS corresponding to 58.75 g/kg flour) was obtained in Q LS at DY 250 in the presence of 6% sucrose after a shorter fermentation time (15 h). Interesting results on the rheological and textural properties of wheat and sorghum sourdough breads were obtained by Galle et al. (2012a,b) after addition (10 or 20% on dough weight) of EPS-enriched sourdough containing from 0.6 to 8 g/kg of EPS. Therefore, to obtain an improvement in bread quality, it can be enough to add the 1% of the C43-11 quinoa based LS (DY250, 6% sucrose) or the 10% of the 21B W/Q LS DY250 and 3% sucrose, to the bread dough.

The higher EPS production by C43-11 in LSs at DY 250 with 6% sucrose could be related to the osmotic stress generated by the presence of a high content of sucrose (Rühmkorf et al., 2012). In our study, by reducing the DY and therefore the water content, the TTA values increased, mainly in the presence of the pseudocereal alone, indicating a higher amount of undissociated acids, while the pH of inoculated LSs, slightly decreased and the content of lactic and acetic acids remained almost unvaried. The pH increase observed at lower DY (250) could be related to the lower water content and consequently to the lower diffusion of organic acids in the environment (De Vuyst et al., 2014). Moreover, in the presence of quinoa higher pH values were observed indicating the buffering capacity of the pseudocereal as suggested by other authors (Rühmkorf et al., 2012). The slower acidification observed in LSs at DY 250 could be responsible for the lower protein hydrolysis registered for these samples. In fact, as reported by Spicher and Stephan (1999), at higher DY organic acids easily diffuse into the environment and rapidly acidify it. Whereas, in sourdough with low DY the higher amount of carbohydrates available for fermentation lead to a higher buffering capacity that slows the acidification rate (De Vuyst et al., 2014). Moreover, in the presence of higher amounts of EPS the proteolysis lowered maybe since the ability of the polysaccharides to form a network hampering the hydrolysis of proteins, mainly the soluble ones: this can also be the reason of the higher pH (about 4.3) observed in C43-11 LSs at DY 250 respect to that of LS at DY500 (3.95÷4.04). However, an appreciable protein hydrolysis and EPS production was observed with the strain ITM21B, mainly in LSs at DY250 and sucrose 6%. The *L. plantarum* strain was selected during the screening test in mMRS_S as not-producer strain, but it became able to produce EPS by modifying the LS formulation ensuring a good proteolysis and therefore having a key role in improving the overall quality of sourdough bread (Gobbetti et al., 2019). In particular, proteolysis is a fundamental process in sourdough technology of gluten free bread since it positively affects the quality of final products, as reported by several authors (Moroni et al., 2009; Coda et al., 2010; Dallagnol et al., 2013; Rizzello et al., 2017). Generally, in our study a higher protein degradation was observed for both strains in LSs containing only the pseudocereal maybe for the more hydrophilic nature of their proteins which can be quickly degraded (Dallagnol et al., 2013; Wolter et al., 2014).

The LoaC capillary electrophoresis offers great advantages over classic SDS-PAGE and, due to the improved resolution of peaks, provided quantitative and reproducible results on the protein profile changes after LAB fermentation. The protein profile and overall arrangement of bands in wheat, quinoa and amaranth flours well-compared with previous studies in which the same protein profiles were observed on Protein 230 Loac or SDS gels (Hager et al., 2012; Alonso-Miravalles and O’Mahony, 2018). Wheat flour was mainly characterized by the presence of protein bands in the 43–55 kDa range which could represent gliadins and the low molecular weight glutenins (Bradová and Matějová, 2008; Juhász et al., 2015). In amaranth and quinoa flours, the presence of the typical proteins amaranthin and chenopodin, respectively, was ascertained in the A1 and A2 areas which represent the region generally modified during fermentation (Lacaze et al., 2007). Actually, the most relevant changes in LSs fermented by C43-11 and ITM21B strains occurred in those areas where an increase of low molecular weight proteins was observed. An interesting result was the consistent degradation of proteins in the 43–55 kDa region, containing the gliadin and glutenin fractions of wheat, observed in W/Am and W/Q LSs at DY 250 (3 or 6% sucrose).

## Conclusion

Results demonstrated that the modulation of fermentation parameters (flour type, DY, sucrose) can stimulate metabolic activities of *W. cibaria* C43-11 and *L. plantarum* ITM21B making them suitable for the production of short fermented (15 h) LSs, enriched in the EPS content, which can be applied in the bread-making process. The *W. cibaria* strain (C43-11), isolated from Italian wheat semolina, was able to produce a considerable amount of EPS mainly in the presence of pseudocereals amaranth or quinoa and to hydrolyze proteins during fermentation. A more efficacious proteolysis with degradation of proteins in the region including the glutenin and gliadin fraction of wheat, was obtained using the *L. plantarum* sourdough strain ITM21B in the wheat/quinoa formulations at DY 250.

In conclusion, the study highlighting the still unexplored potential of LAB strains in food manufacturing and their contribution in improving the rheological and functional features of bakery products.

## Data Availability Statement

The raw data supporting the conclusions of this article will be made available by the authors, without undue reservation.

## Author Contributions

FV conceived and supervised the study. FV, MD, AB, and SL designed the experiments and wrote the first draft of the manuscript. MD, AB, and SL performed the experiments. PL coordinated the research. AL coordinated the funding projects. PL, FV, and SL provided a critical revision of the manuscript. All authors interpreted the results, contributed to the manuscript revision, and read and approved the submitted version.

## Conflict of Interest

The authors declare that the research was conducted in the absence of any commercial or financial relationships that could be construed as a potential conflict of interest.
