# Supplementary material for: Effect of Amaranth and Quinoa Flours on Exopolysaccharide Production and Protein Profile of Liquid Sourdough Fermented by Weissella cibaria and Lactobacillus plantarum
Source: Front Microbiol. 2020 May 21;11:967. doi: 10.3389/fmicb.2020.00967 (PMC7253592; doi:10.3389/fmicb.2020.00967)
Supplement: Supplementary file 1 [file Data_Sheet_1.DOCX]

**Table S1**. EPS production by 21 LAB strains screened on mMRS agar plates containing different carbon sources: glucose (G), fructose (F) or sucrose (S)

| **Strain** | **mMRS_G** | **mMRS_F** | **mMRS_S** |
| --- | --- | --- | --- |
| *L. brevis* 18F | - | - | - |
| *L. hilgardi* 51B | - | - | - |
| *L. paracasei* IMPC 2.1 | - | - | - |
| *L. paracasei* IMPC 4.1 | - | - | - |
| *L .pentosus* 15BG | - | - | - |
| *L. pentosus* 14TG | - | - | - |
| *L. plantarum* ITM21B | - | - | - |
| *L. plantarum* 19A | - | - | - |
| *L. sanfranciscensis* C57 | - | - | - |
| *Ln. citreum* C2-27 | - | - | +/- |
| *Ln. mesenteroides* C43-18 | - | - | + |
| *W. cibaria* C21-4 | - | - | + |
| *W. cibaria* C2-5 | - | - | + |
| *W. cibaria* C43-11 | - | - | ++ |
| *W. cibaria* C3-2 | - | - | ++ |
| *W. cibaria* C3-4 | - | - | ++ |
| *W. cibaria* C3-19 | - | - | ++ |
| *W. cibaria* C4-21 | - | - | ++ |
| *W. cibaria* C2-32 | - | - | ++ |
| *W. confusa* C5-7 | - | - | ++ |
| *W. confusa* C5-4 | - | - | ++ |

**Table S2.** Total protein content (mg/g of flour) of optimized LSs as measured by the Bradford assay at time 0.

| **LS Flour** | **Total proteins**  **(mg/g flour)** | | |
| --- | --- | --- | --- |
|  | DY500_3%S | DY250_3%S | DY250_6%S |
| W/Am | 20.28 ± 1.38ab | 17.56 ± 2.17ab | 19.25 ± 0.96ab |
| W/Q | 16.67 ± 0.39ab | 16.53 ± 0.54ab | 15.20 ± 0.27a |
| Am | 21.81 ± 0.71b | 18.26 ± 1.47ab | 18.71± 0.92ab |
| Q | 19.47 ± 0.82ab | 16.60 ± 1.78ab | 16.40 ± 1.96ab |
